# Decreased expression of SRY-box containing gene 30 is related to malignant phenotypes of human bladder cancer and correlates with poor prognosis

**DOI:** 10.1186/s12885-018-4560-x

**Published:** 2018-06-07

**Authors:** Yang Liu, Han Wang, Jianhua Zhong, Chenglong Wu, Gang Yang, Yuantang Zhong, Jinghua Zhang, Aifa Tang

**Affiliations:** 1 The Central Laboratory, Shenzhen Second People’s Hospital, Graduate School of Guangzhou Medical University, Shenzhen, China; 2 Department of Urinary Surgery, Shenzhen Second People’s Hospital, The First Affiliated Hospital of Shenzhen University, Shenzhen, China

**Keywords:** Bladder cancer, SOX30, Proliferation, Invasion, Apoptosis, Therapeutic target

## Abstract

**Background:**

In human pulmonary malignancies, the SRY-box containing gene 30 (*SOX30*) is a known cancer-suppressing gene. Nevertheless, its molecular role and clinical effects remains unknown in bladder cancer.

**Methods:**

*SOX30* mRNA expression was quantified in bladder cancer tissue, paired adjacent normal tissue, and cell lines with qRT-PCR. *SOX30* protein expression in BC tissue and cell lines was evaluated via western blotting and immunohistochemistry. In addition, the clinical and prognostic significance of *SOX30* in BC were assessed using Kaplan-Meier analysis. Furthermore, we measured cell migration and invasion, cell proliferation and cell apoptosis by means of a Transwell assay, cell counting kit-8 along with flow cytometry, respectively.

**Results:**

Expression levels of *SOX30* were markedly lower in BC cells and tumor tissues than in adjacent noncancerous tissues. Moreover, clinicopathological analyses showed that low *SOX30* expression was positively related to an advanced tumor, node, and metastasis (TNM) stage. Furthermore, low *SOX30* expression conferred reduced survival rates (*P* < 0.05). Functional analyses revealed that *SOX30* overexpression attenuated cell proliferation, invasion, and migration, while promoting apoptosis in BC cells.

**Conclusions:**

*SOX30* displays tumor suppressive behavior, warranting future investigations into its therapeutic potential in the treatment of BC.

## Background

Bladder cancer (BC) lays claim to being the fifth most common carcinoma, representing a genitourinary tract tumor that occurs most frequently in men within developed countries [1–4]. BC may be clinically categorized into muscle-invasive BC (MIBC) or non-muscle-invasive BC (NMIBC) [5]. An estimated 70% of NMIBC patients have a high recurrence rate (50–70%) after transurethral resection, and approximately one-third of patients diagnosed with BC will progress to metastatic disease [3, 5–7]. Although improvements in therapeutic methods and drugs have been implemented in recent years, the overall survival rate of BC patients has not observably improved because of the high rate of metastasis and recurrence [2, 4, 8, 9]. Therefore, there is an urgent need to explore new tumor markers and therapeutic targets for BC.

The Y chromosome contains a mammalian sex determining region Y (*SRY*) gene that contains instructions for synthesizing a transcription factor with the HMG-box region, DNA-binding domain of 79 amino acids in length [10–12]. Based on sequence similarity to the HMG domain of *Sry*, at least 50% of Sox family members have been identified [13, 14]. Numerous earlier studies have shown that Sox proteins are essential for embryogenesis and development, including biological sex differentiation and determination, testicular development, and maintenance of male fertility [15].

According to previous research, the expression of *SOX30*, a member of the Sox category of proteins, is associated with spermatogonial differentiation and spermatogenesis functions in mice and humans [14, 16]. *SOX30* members modulate genetic expression controlling a myriad of processes related to development; however, the regulation may occur at different stages and differ according to sex [13, 17]. In lung cancer, *SOX30* is currently known to be downregulated and affects cellular apoptosis by transcriptionally activating p53 [18]. However, the biological function and clinical significance of *SOX30* in BC remain unclear. Our investigations seek to explore how *SOX30* is expressed in BC along with its biological roles in regulating cell migration, proliferation, and apoptosis in BC.

## Methods

### Patient samples

In this study, 30 pairs of BC tissue and normal paracancerous tissue were gained from Zhujiang Hospital (Guangdong, China) and quickly exposed to liquid nitrogen to stimulate freezing post-resection.

### Bladder cancer cell lines

Human BC lines for research:5637(catalog number: ATCC® HTB-9™), T24(catalog number: ATCC® HTB-4™), SW780(catalog number: ATCC® CRL-2169™), and J82(catalog number: ATCC® HTB-1™) and normal bladder cells SV-HUC-1(catalog number: ATCC® CRL-9520™) were gained from the American Type Culture Collection. SW780 and 5637 cells were maintained in RPMI 1640 medium, T24 cells in 5A medium, J82 cells in Minimum Essential Medium and SV-HUC-1 cells in F-12 K medium; all culture media contained 10% fetal bovine serum (FBS, Gibco, Australia).

### Extraction of RNA and qRT-PCR

Cancer cell lines and tumor tissue specimens were subjected to RNA extraction with TRIzol reagent (Ambion) using instructions provided in the product manual. cDNA (20 μl) was produced with the help of ReverTra Ace qPCR RT master mix (Toyobo, Japan). The reaction containing 1 μg of RNA was maintained for 15 min at 37 °C, and then for 5 min at 50 °C and another 5 min at 98 °C followed by exposure to 4 °C for the remainder of the run. A relative quantitative analysis was performed to determine mRNA expression in tissue samples or cultured cells using a RT-PCR and SYBR Green method. All gene expressions were normalized to β-Actin. Primer sequences are as follows: *SOX30* 5′ CCAAGCCCTGTCACACTTTT 3′(forward) and 5′ AATCCTGTTGGCGCTCTCTA 3′(reverse); β-actin 5′ CAATGACCCCTTCATTGACC 3′(forward) and 5′ GACAAGCTTCCCGTTCTCAG 3′(reverse). The comparative threshold cycle (CT) method was used to calculate the relative mRNA expression levels of *SOX30*.

### Western blot analysis

BC cells and BC tissue samples were rinsed with phosphate-buffered saline (PBS) on ice. An appropriate amount of radioimmunoprecipitation assay (RIPA) buffer (Pierce) mixed with protease inhibitor (1:100 dilution, Thermo scientific, USA) was added. A bicinchoninic acid (BCA) protein assay kit (Pierce) was then used to detect total protein concentrations. Samples were electrophoretically run on a 12% polyacrylamide gel, and then proteins (20 μg per sample) were applied onto a polyvinylidene difluoride (PVDF) membrane (Millipore, Germany). Protein samples on the membranes were incubated with anti-*SOX30* antibodies (1:1500, Santa Cruz Biotechnology, USA) for 60 min and anti-β-tubulin antibodies (1:8000, Abcam, UK-E) overnight at 4 °C along with a small vibration. The following morning, membranes were TBST-rinsed and left for a final incubation with goat anti-rabbit secondary antibodies (1:8000, Abcam, UK-E) for 1 h on the basis of the internal control. Chemiluminescence imaging instruments were provided by Gene Company Limited.

### Culture of stable cell lines

A lentivirus vector was used to clone full-length *SOX30* cDNA along with negative controls (Gene Pharma, China). For transduction, lentiviral constructs expressing *SOX30* or the negative control were transduced into 5637 and T24 cells, respectively. *SOX30* expression levels were identified via western blot and qRT-PCR.

### Cell proliferation

Quantification of cell proliferation was carried out utilizing a CCK-8 assay (CCK-8, Dojindo, Kumamoto, Japan). T24 or 5637 cells (100 μl, 2 × 103 cells) were planted onto 96-well plates. After 24 h, CCK-8 solution (10 μl) was inserted into 5 wells in the overexpression and negative control groups. Cell proliferation assay was performed according to our previous study [5]. Results were obtained for the overexpression groups and negative control group at different time points (0–4 days) in three independent trials via detection at 450 nm absorbance.

### Cell migration and invasion assays

To determine the capabilities T24 or 5637 cells to migrate, Transwell chambers were used to conduct the experiment. Approximately 3 × 104 transduced cells in 300 μl of medium without FBS were loaded onto the higher chamber, with 500 μl of medium containing 10% serum placed in the bottom slot. The operation of both the cell migration and invasion assays were similar was similar; however, invasion-related experiments utilized a chamber coated with Matrigel, and then the transduced T24 or 5637 cells were allowed to migrate or invade for 24 h. Bladder cancer cells on the upper chambers were gently eliminated, and cells found on the bottom-most surface were subjected to fixation with 4% paraformaldehyde for 25 min. Crystal violet (0.05%) was used to stain migratory cells. The migration ability of the cells was summed from images of five random microscopic fields per well.

### Cellular apoptosis analysis

Transduced cells were digested using trypsin, centrifuged at 2000 rpm for 3 min, and then resuspending transduced cells (1 × 106) in 100 μl of 1 × binding buffer, which contained and 5 μl of PI and 5 μl of annexin-V- FITC. A 10–15 min incubation in a dark room was carried out for all transduced cells, in accordance to detailed steps described in our previous study [5]. The samples were analyzed via flow cytometry and subjected to three experimental repetitions.

### Immunohistochemistry (IHC) analysis

A paraffin-embedded BC tissue microarray, including 56 pairs of cancerous tissues and 10 pairs of adjacent tissues, was purchased from the Shanghai Biochip Company Ltd. (Shanghai, China). Antigen retrieval with a sodium citrate solution (10 mM, pH 6.0) was performed at a high temperature for 2 min, a low temperature for 10 min twice, and then at room temperature. Samples were then incubated in a 3% hydroxyl peroxide solution for 10–15 min to reduce nonspecific background staining attributable to endogenous peroxidase; sheep serum was then added for 30 min to block non-specific background staining after washing with PBS twice for 5 min. After the addition of an anti-*SOX30* antibody (1:200), samples were left overnight at 4 °C. A 30 min incubation at 24 °C followed the next day. Finally, samples were incubated with a biotin-labeled goat anti-rabbit antibody for 30 min, colored with 3,3′-diaminobenzidine and hematoxylin-stained. The dyeing times were obtained by observing the extent of color development under a microscope.

### Statistical analysis

Statistically significant differences between BC tissue and para-carcinoma tissue were determined using a paired samples t-test with SPSS 19.0 (SPSS, USA). Analysis of variance (ANOVA) and independent-samples t-test were employed for CCK-8 data analysis and other experimental results, respectively. Chi-squared analysis allowed us to assess the relationship between *SOX30* expression and the clinicopathological characteristics of BC. *P* < 0.05 indicated a statistical significance.

## Results

### *SOX30* expression found to be suppressed in human BC tissue and BC cell lines

**Fig. 1:**
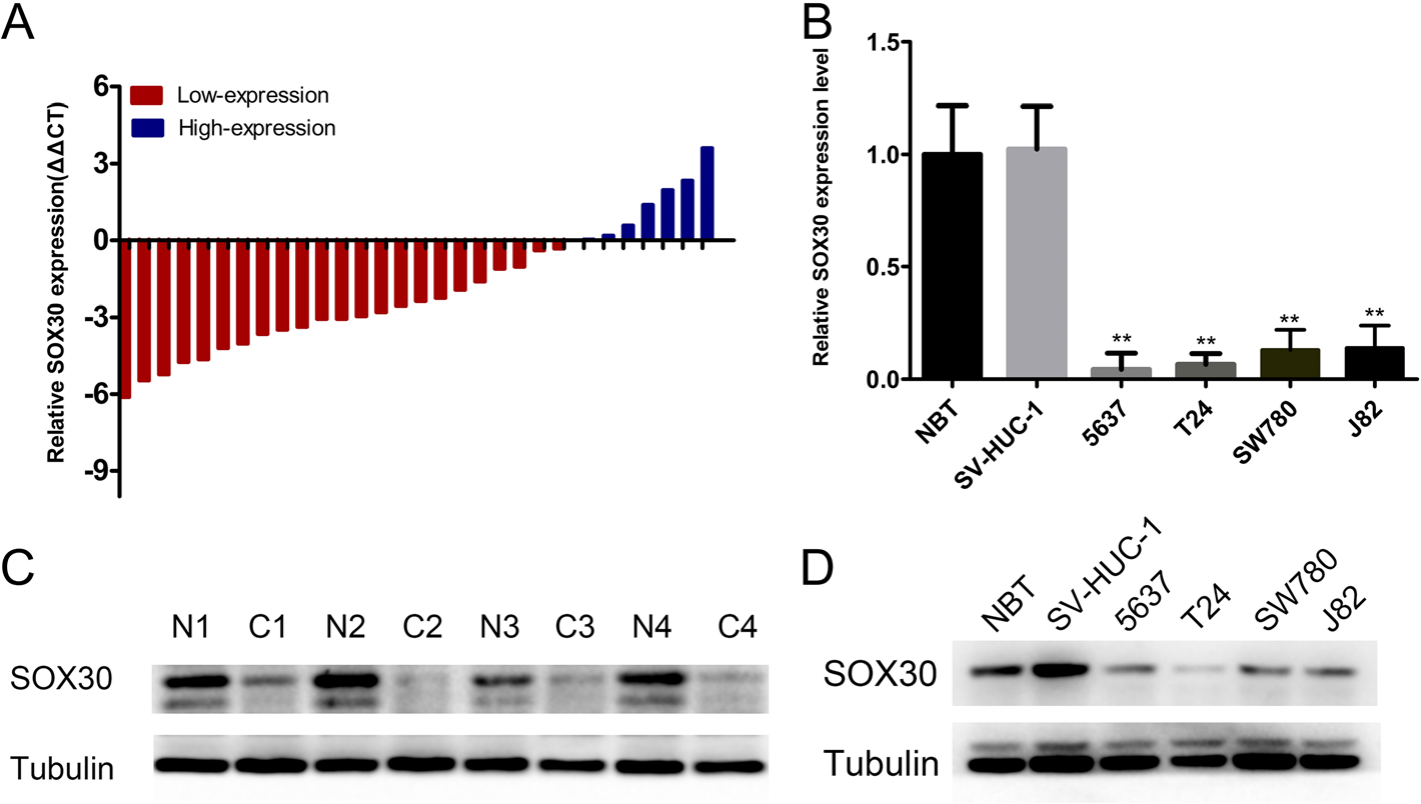
SRY-box containing gene 30 (*SOX30*) was downregulated in bladder cancer. Western blotting and real-time quantitative PCR (qRT-PCR) were utilized to quantify *SOX30* expression levels. **a** Bladder cancer tissues had decreased relative *SOX30* expression levels. **b** qRT-PCR revealed that SV-40-immortalized human uroepithelial cells and normal bladder tissues (NBT) had higher *SOX30* expression levels compared to T24 and 5637 bladder cancer cell lines. Data is depicted in terms of mean ± SD.**P < 0.01. **c** Western blotting revealed that pair-matched adjacent normal bladder tissues had higher *SOX30* expression levels compared to bladder cancer tissues. **d** Western blotting uncovered that SV-40-immortalized human uroepithelial cells and NBT had higher *SOX30* expression levels compared to T24 and 5637 bladder cancer cell lines

To determine in vitro *SOX30* expressions, 30 BC tissue pairs and adjacent tissues were examined for RNA and protein levels. qRT-PCR results suggested that *SOX30* expression was significantly lower in 80% (23/30) of the BC tissues than in adjacent cancer tissue (Fig. 1a). We selected 5 pairs of BC tissue and their corresponding adjacent tissue to measure protein expression via western blotting. *SOX30* was expressed to a lower degree in BC tissues in contrast to healthy bladder tissues adjacent to the tumor. Western blot results were consistent with RNA levels (Fig. 1c). Furthermore, we determined the RNA and protein levels of *SOX30* in cell lines. *SOX30* expression was significantly lower in BC cells (Fig. 1b and d, **P < 0.01) compared to SV-HUC-1 cells and normal bladder tissue.

### Low expression of *SOX30* conferred worse patient prognosis in those with BC

**Fig. 2:**
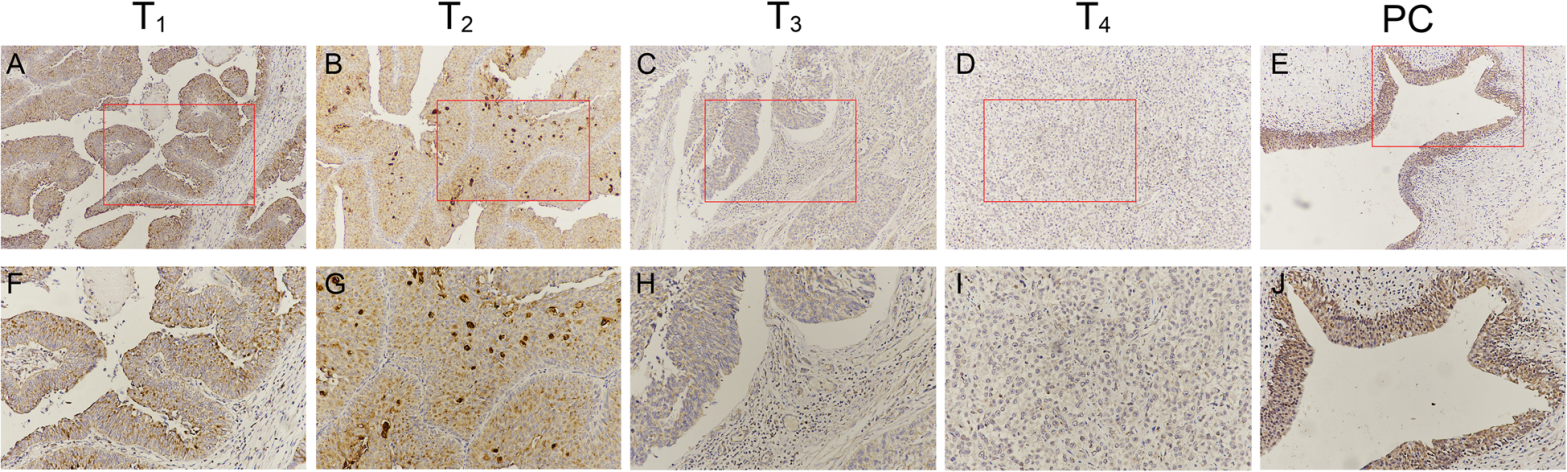
SRY-box containing gene 30 (*SOX30*) expression levels in patients with different tumor, node, and metastasis (TNM) stages of bladder cancer: T 1 (**a**, **f**); T 2 (**b**, **g**); T 3 (**c**, **h**); T 4 (**d**, **i**); **e**, **j** positive *SOX30* staining (positive control, PC). 100 μm scale bar for **a**–**i**; 50 μm scale bar for **f**–**j**

**Fig. 3:**
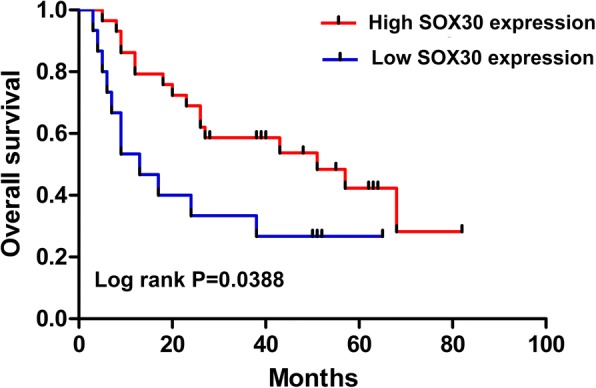
Patients with higher levels of SRY-box containing gene 30 (*SOX30*) expression showed longer overall survival times than patients with lower levels of *SOX30* expression (log-rank *P* < 0.05)

IHC staining revealed an elevated *SOX30* protein expression in healthy bladder epithelium, and conversely a relatively low expression in BC tissues (Fig. 2). To determine the clinical significance of these molecular differences, further analysis was performed in efforts to correlate clinicopathological features to *SOX30* expression. As shown in Table 1, downregulation of *SOX30* was significantly related to advanced tumor, node, and metastasis (TNM) stages (*P* = 0.019, Table 1), but not to age, sex, tumor size, clinical grade, or pathological type. Overall survival (OS) calculations as evaluated via log-rank tests and Kaplan-Meier curves revealed that a suppressed expression of *SOX30* tended to yield poorer patient prognosis (*P* = 0.0388) (Fig. 3).

**Table 1:** Correlation between SOX30 expression and clinicopathological characteristics of bladder cancer patients

Characteristic	case number(*n* = 56)	SOX30 expression		*P*-value
		High (*n* = 35)	Low (*n* = 21)	
Gender				0.639
Male	47	30	17	
Female	9	5	4	
Age				0.093
≥ 60 years	44	30	14	
<60 years	12	5	7	
TNM stage				0.019^a^
T1-2	30	23	7	
T3–4	26	12	14	
N status				0.138
N_0_	50	30	20	
N_1–3_	6	5	1	
Histologic grade				0.068
G_1–2_	19	15	4	
G_3–4_	37	20	17	
Tumor size				0.150
≥ 5 cm	20	15	5	
< 5 cm	36	20	16	
Pathological type				0.317
Urothelium carcinoma	33	18	15	
Papillary carcinoma	13	11	2	
Squamous-cell carcinoma	5	4	1	
Glandular carcinoma	5	2	3	

^a^Statistically significant

### Generation of cell lines overexpressing *SOX30*

**Fig. 4:**
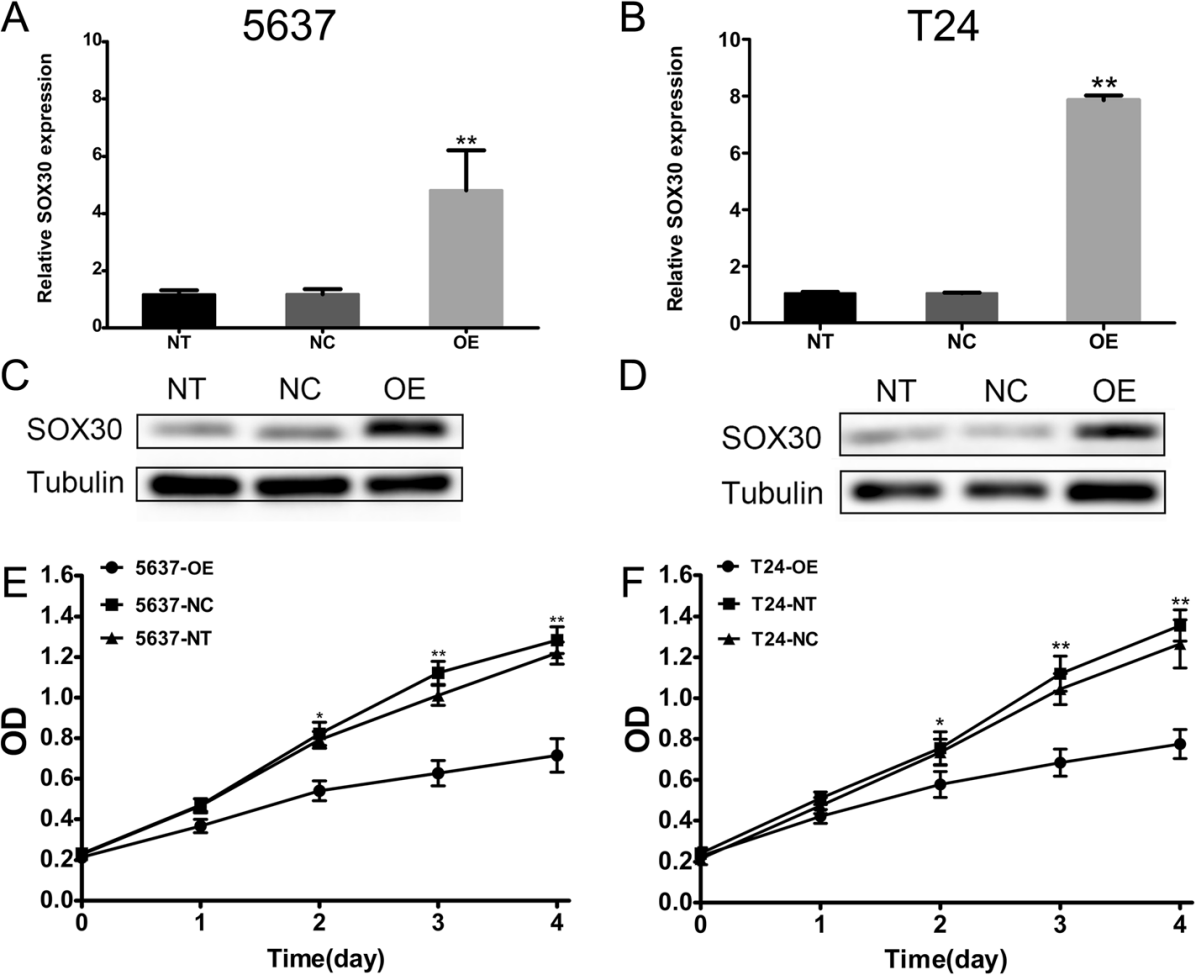
SRY-box containing gene 30 (*SOX30*) is overexpressed in T24 and 5637 bladder cancer cell lines. Western blot and qRT-PCR analyses of *SOX30* from empty vector-transduced control (NC), non-transduced control (NT) and target gene-transduced cells (OE) are depicted for 5637 (**a**, **c**) and T24 (**b**, **d**) cells. Overexpression of SRY-box containing gene 30 (*SOX30*) inhibited proliferation of bladder cancer cell, as revealed via a Cell Counting Kit-8 (CCK-8) assay. Inhibition of cellular proliferation was observed in 5637 (**e**) and T24 (**f**) bladder cancer cells. Data is depicted in terms of mean ± SD. (*P < 0.05, **P < 0.01)

We sought to extend current knowledge of *SOX30*’s function in BC by generating *SOX30*-overexpressing T24 and 5637 cell lines. As shown in Fig. 4a–d, overexpression of *SOX30* in these cell lines was successful. Altered T24 and 5637 cells were found to have clearly elevated *SOX30* mRNA and protein levels than in the empty vector-transduced control (NC) group.

### Overexpression of *SOX30* suppresses BC cell proliferation

Further cell proliferation studies via CCK-8 assays that were done to confirm how *SOX30* expressions influenced cell activity revealed that both T24 and 5637 cell lines with high *SOX30* expression had a lower proliferation rate than the T24-NC and 5637-NC groups (Fig. 4e–f).

### Overexpression of *SOX30* inhibits BC cell migration and invasion

**Fig. 5:**
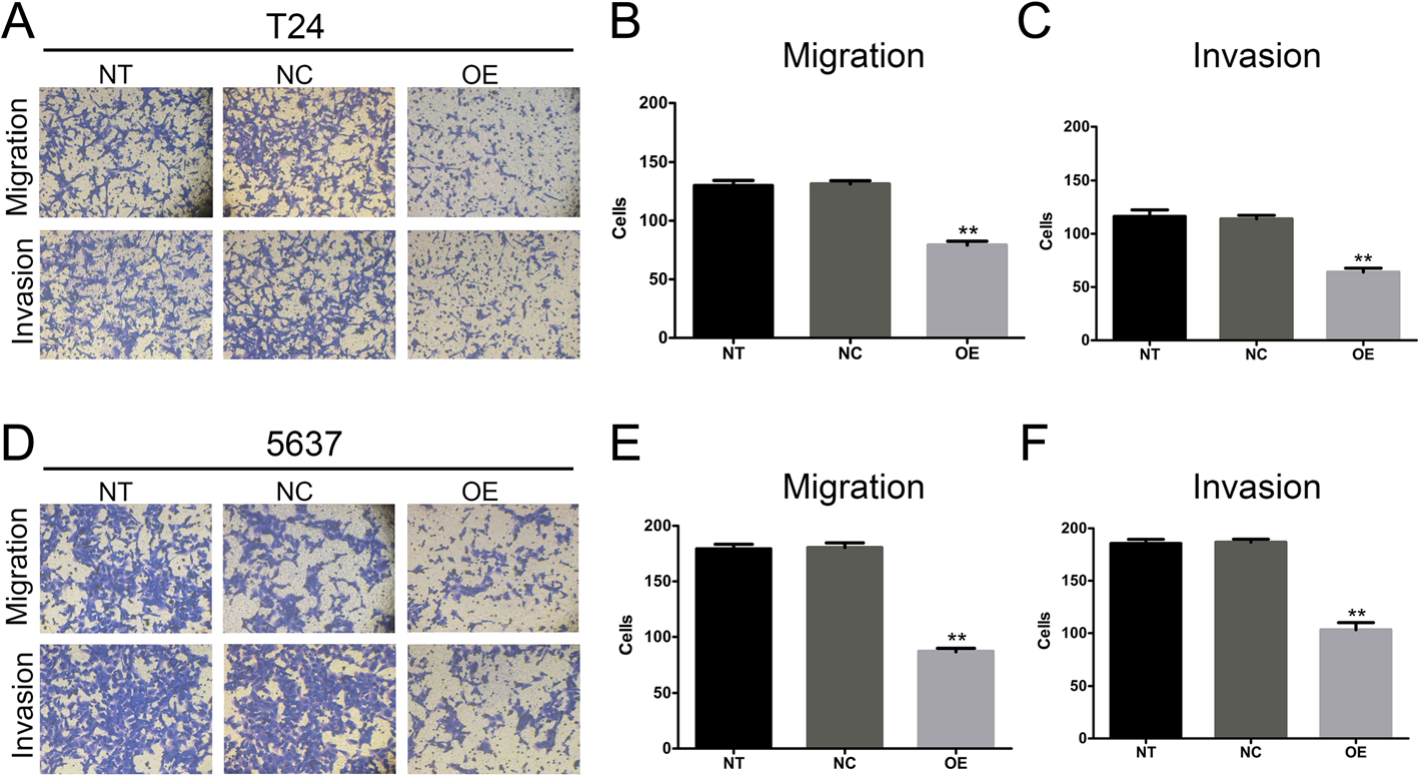
SRY-box containing gene 30 (*SOX30*) inhibits 5637 and T24 bladder cancer cell invasion and migration. **a**–**c** *SOX30* overexpression and its effects on T24 cell invasion and migration are shown. **d**–**f** *SOX30* overexpression and its effects on 5637 cell invasion and migration are shown. Data is depicted in terms of mean ± SD. **P < 0.01. Each assay was performed in triplicate. NT, non-transduced control; NC, empty vector-transduced control; OE, target gene-transduced cells

How *SOX30* affected BC cell invasion and migration was investigated with Transwell assays. *SOX30* overexpression significantly inhibited migration of 5637 and T24 cell lines (Fig. 5a and b, d and e). Similarly, Matrigel invasion assays indicated that *SOX30* overexpression suppressed the invasion ability of T24 and 5637 cells. Our findings demonstrate the ability of *SOX30* to attenuate cell invasion and migration in BC cells (Fig. 5a and c, d and f).

### Overexpression of *SOX30* increases apoptosis in T24 and 5637 cells

**Fig. 6:**
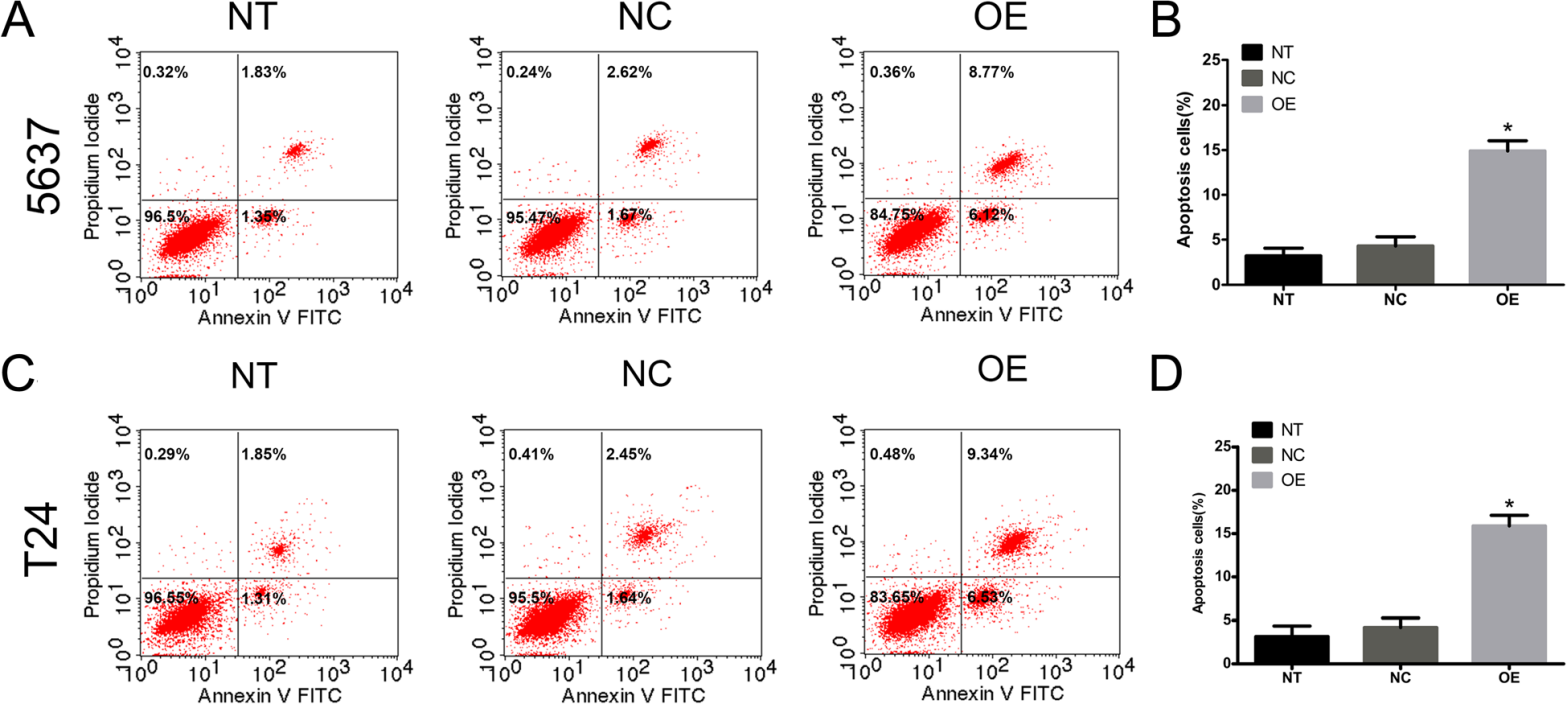
Overexpression of SRY-box containing gene 30 (*SOX30*) induces apoptosis in 5637 and T24 cells. Cellular apoptosis was induced in bladder cancer 5637 (**a**, **b**) and T24 (**c**, **d**) cells. Data is depicted in terms of mean ± SD. **P < 0.01. Each experiment underwent three repetitions under independent conditions

As previously described, *SOX30* inhibits BC cell proliferation. A flow cytometric analysis was conducted to explore how *SOX30* affected cellular apoptosis. These experiments demonstrated that *SOX30*-overexpressing cells experienced elevated apoptotic rates compared to negative control 5637 and T24 cells (Fig. 6). Our findings demonstrate the ability of *SOX30* to induce apoptosis in BC cell lines in vitro.

## Discussion

BC is a very serious health issue worldwide, with relatively high morbidity and mortality rates [19]. Therefore, an understanding of the molecular mechanisms and biological functions of BC development is imminently needed to improve prognosis and treatment outcomes.

To date, approximately 30 *SOX* genes encoding proteins containing the HMG domain have been found based on structure, organization, similarity, and other characteristics. There are 10 families of genes related to SOX, designated A to J [10, 20, 21]. SOX30 is located on human chromosome 5 (5q33) and belongs to the H group; it was initially extracted from mice and humans. Studies suggest that SOX30 exists in both mammals and non-mammals, and it is considered a gonad-specific gene associated with stage and phenotypic sex [13, 14]. Furthermore, diminished methylation at CpG islands in *SOX30* could promote *SOX30* expression in mouse developmental testes, and *SOX30* expression can be restored by 5-aza-2′-deoxycytidine in TM4 (Sertoli), TM3 (Leydig) and GC2 (GC-2 spd - spermatocyte), cell lines [15]. *SOX30* expression is downregulated in the human trabecular meshwork via triamcinolone acetonide and dexamethasone treatment [22]. Moreover, *SOX30* is silenced by hypermethylation and has been found in lung cancer; *SOX30* overexpression inhibits lung cancer cell proliferation, induces cellular apoptosis in vitro, and represses tumor formation in vivo. In addition, the antitumor effects of *SOX30* result from attachment to the CACTTTG (+ 115 to + 121) region of the p53 promoter, acting as a new transcriptional activating factor of p53 [18]. *SOX30* also preferentially activates p53 transcription at the ACAAT motif [14]. In human lung adenocarcinomas, *SOX30* expression correlates well with the histological type as well as lymphatic metastasis; high *SOX30* expression is related to favorable survival [23]. Recently, Guo et al. [24] observed that SOX30 may act as a miR-645 target in colon cancer.

The present study shows that *SOX30* expression is considerably lower in BC than in adjacent normal tissues and that poor survival in BC as well as advanced TNM stages are significantly linked to lower *SOX30* expression (*P* < 0.05 for both). Furthermore, healthy bladder tissue and normal bladder cell lines (SV-HUC-1 cells) expressed higher *SOX30* expression in contrast to levels found in BC cell lines. Interpreted as a whole, these findings allude towards *SOX30*’s role in BC tumorigenesis. To discover the significance of *SOX30* in BC, we examined cell apoptosis, invasion, migration as well as proliferation of BC cell lines T24 and 5637 modified to overexpress *SOX30* using a lentiviral vector. The results show that overexpression of *SOX30* significantly inhibited cell invasion, migration as well as proliferation while promoting apoptosis in T24 and 5637 cells. As such, overexpression of *SOX30* could repress the progression and development of BC. However, the present study is limited in terms of the number of BC tissue samples and the number of paraffin-embedded bladder cancer tissue samples used in the microarray; therefore, further studies should verify these results using a larger case series. Moreover, this study was just a preliminary analysis, and deeper gene interactions and related signaling pathways need to be further explored.

## Conclusion

This experiment demonstrates that BC cells express downregulated levels of *SOX30*, a phenomenon related to poor prognosis and advanced TNM stage. Additionally, *SOX30* was also discovered to be a key driver of proliferation, invasion, migration, and apoptosis of BC cells, suggesting the tumor suppressive function of *SOX30.* This gene should be further investigated for its prognostic potential as well as its ability to serve as a therapeutic target in treating BC.

## Abbreviations

BC: Bladder cancer
CCK-8: Cell Counting Kit − 8
MIBC: Muscle-invasive bladder cancer
NMIBC: Non-muscle-invasive bladder cancer
SOX30: SRY-box containing gene 30
TCC: Transitional cancer cells
TURBT: Transurethral resection of the bladder tumor
